# Stoichiometric Modeling of Artificial String Chemistries Reveals Constraints on Metabolic Network Structure

**DOI:** 10.1007/s00239-021-10018-0

**Published:** 2021-07-06

**Authors:** Devlin Moyer, Alan R. Pacheco, David B. Bernstein, Daniel Segrè

**Affiliations:** 1 Bioinformatics Program, Boston University, Boston, MA 02215 USA; 2 Department of Biology, Boston University, Boston, MA 02215 USA; 3 Biological Design Center, Boston University, Boston, MA 02215 USA; 4 Department of Biomedical Engineering, Boston University, Boston, MA 02215 USA; 5 Department of Physics, Boston University, Boston, MA 02215 USA

**Keywords:** Artificial chemistry, Flux balance analysis, Metabolism, Genome-scale metabolic models

## Abstract

Uncovering the general principles that govern the structure of metabolic networks is key to understanding the emergence and evolution of living systems. Artificial chemistries can help illuminate this problem by enabling the exploration of chemical reaction universes that are constrained by general mathematical rules. Here, we focus on artificial chemistries in which strings of characters represent simplified molecules, and string concatenation and splitting represent possible chemical reactions. We developed a novel Python package, ARtificial CHemistry NEtwork Toolbox (ARCHNET), to study string chemistries using tools from the field of stoichiometric constraint-based modeling. In addition to exploring the topological characteristics of different string chemistry networks, we developed a network-pruning algorithm that can generate minimal metabolic networks capable of producing a specified set of biomass precursors from a given assortment of environmental nutrients. We found that the composition of these minimal metabolic networks was influenced more strongly by the metabolites in the biomass reaction than the identities of the environmental nutrients. This finding has important implications for the reconstruction of organismal metabolic networks and could help us better understand the rise and evolution of biochemical organization. More generally, our work provides a bridge between artificial chemistries and stoichiometric modeling, which can help address a broad range of open questions, from the spontaneous emergence of an organized metabolism to the structure of microbial communities.

**Supplementary Information:**

The online version contains supplementary material available at 10.1007/s00239-021-10018-0.

## Introduction

Metabolism occupies a central role in the functioning of biological systems, yet much remains unclear about the degree to which basic features of metabolic networks reflect either evolutionary accidents or optimal network structures (Pál et al. 2006; Barve and Wagner 2013; Noor et al. 2010; Ebenhöh and Heinrich 2001). In parallel to analyses focused on metabolism as we know it in individual organisms (Machado et al. 2018; Henry et al. 2010; Borenstein et al. 2008) or in the whole biosphere (Barve and Wagner 2013; Raymond and Segrè 2006; Handorf et al. 2005), multiple studies have explored the utility of abstract models of chemistry to investigate particular features of chemical networks. These models, also known as artificial chemistries, have the benefit of being unconstrained by the limits of what is known about extant metabolism and about its possible intermediate states lost through evolutionary history (Banzhaf and Yamamoto 2015; Benkö et al. 2003; Kauffman 1993).

Artificial chemistry has been used to study various aspects of the origin of life from abiotic chemistry (Guseva et al. 2017; Kauffman 1993; Banzhaf and Yamamoto 2015), common structural features of metabolic networks (e.g., hub metabolites) (Friedlander et al. 2015; Fontana and Buss 1994a, b; Pfeiffer et al. 2005), the general behavior of chemical (not necessarily biochemical) reaction networks (Benkö et al. 2003; Walter Fontana and Buss 1994a, b), the optimality (or lack thereof) of metabolic networks (Riehl et al. 2010; Soyer and Pfeiffer 2010), among other questions (Banzhaf and Yamamoto 2015). The artificial chemistry models used in these studies typically employ highly abstracted representations of chemistry (Riehl et al. 2010; Kauffman 1993; Banzhaf and Yamamoto 2015). However, more precise and realistic models involving either string rules based on formalization of real chemistry (like SMILES (Weininger 1988) and variants thereof (Arús-Pous et al. 2019; Lin et al. 2019)), or de novo approximate quantum mechanics computations (Benkö et al. 2003), have been used to explore the full space of possible real-life chemistry up to a certain degree of complexity (Lee et al. 2019). Artificial chemistry approaches have yielded many insights into general features of metabolism, but these findings have remained largely disconnected from the large body of metabolism research focused on characterizing real metabolic networks. We believe that many novel insights into metabolism will be enabled by combining artificial chemistry with techniques commonly used to study real metabolic networks.

The field of stoichiometric constraint-based modeling has provided many approaches that can be particularly useful for quantitatively understanding the structure and function of metabolic networks (Heirendt et al. 2019; Ebrahim et al. 2013; Gottstein et al. 2016; O’Brien et al. 2015). In particular, Flux Balance Analysis (FBA) is a common technique for studying metabolic networks at the level of a whole organism. FBA estimates the space of possible fluxes through a metabolic network at steady state, and is generally employed to identify metabolic states satisfying some biologically meaningful criterion of optimality (Orth et al. 2010). FBA has been used to simulate multiple types of experiments and phenotypes, such as growth rates and metabolic phenotypes of gene knockouts, growth efficiency on different media, and identification of potential drug targets (Orth et al. 2010; Gu et al. 2019; Kauffman et al. 2003; Yizhak et al. 2015). While FBA and stoichiometric constraint-based modeling have been widely used on the metabolic networks of real organisms, these techniques have only rarely been applied to artificial chemistry networks.

**Fig. 1:**
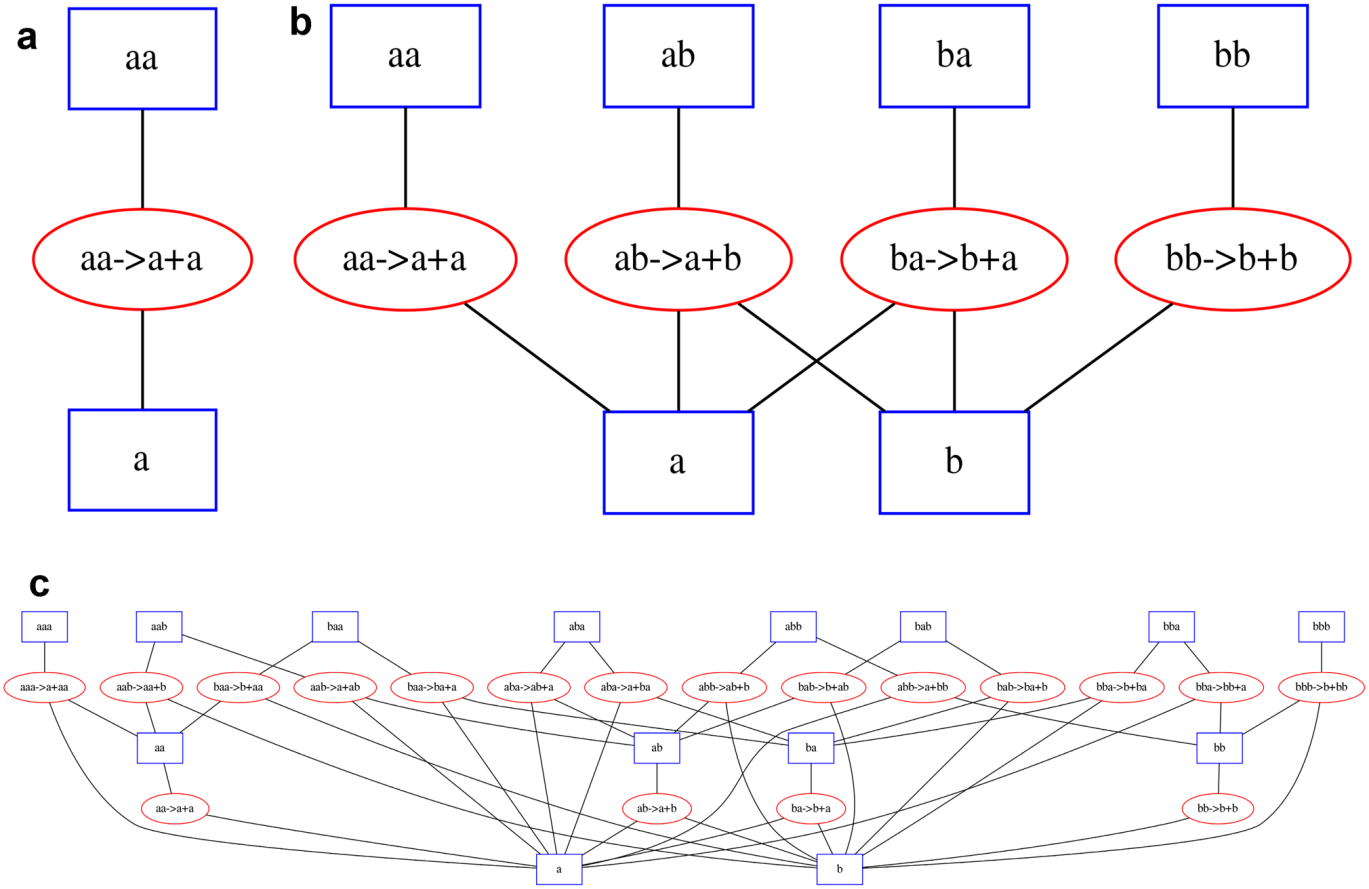
Three simple string chemistry networks. Square nodes represent chemicals and oval nodes represent reactions. Edges connect chemicals to the reactions they participate in, either as reactants or products. **a** A network with only one type of monomer and a maximum string length of 2. **b** A network with two types of monomers and a maximum string length of 2. **c** A network with two types of monomers and a maximum string length of 3

In the present work, we use a specific type of artificial chemistry known as a string chemistry, where each molecule is represented by a string of characters (Fig. 1) (Banzhaf and Yamamoto 2015; Kauffman 1993; Riehl et al. 2010). Our string chemistry model is relatively simple: all strings (i.e., metabolites) are linear sequences of characters (i.e., monomers, atoms, or functional groups) that may react by either concatenating end to end or splitting into two smaller strings (see “Methods” section). A particular string chemistry network is defined by the set of different characters each metabolite can be composed of and the maximum length a metabolite can reach. While these rules are much simpler than those governing real chemical reactions, Riehl et al. found structural similarities between real metabolic networks and string chemistry networks with only one type of character (i.e., the only difference between any two metabolites is their length) (Riehl et al. 2010), so we expect that string chemistries with more than one type of character may yield further insights into the general properties of metabolic networks. In string chemistry networks such as the one we use for this work, individual monomers (the letters in strings) could be thought of as elementary moieties (either atoms, or functional groups). While individual monomers cannot turn into each other (e.g., a letter “a” cannot transform into a letter “b”), one can think of strings such as “ab” and “ba” as more complex functional units that can transform into each other through a series of reactions.

In this manuscript, we describe the ARtificial CHemistry NEtwork Toolbox (ARCHNET), a Python package for generating string chemistry networks of arbitrary size and implementing stoichiometric modeling algorithms (including FBA) on those networks. Using this string chemistry framework, we created an algorithm for determining the minimal metabolic network capable of producing a given set of metabolites (“biomass precursors”) from another set of metabolites (“environmental nutrients”). Our analysis of random choices of nutrients and biomass precursors in different string chemistry networks provides new insight into the rules governing which reactions are left in these minimal metabolic networks and suggests possible implications for the study of real metabolic networks.

## Methods

### Artificial Chemistry Model

**Fig. 2:**
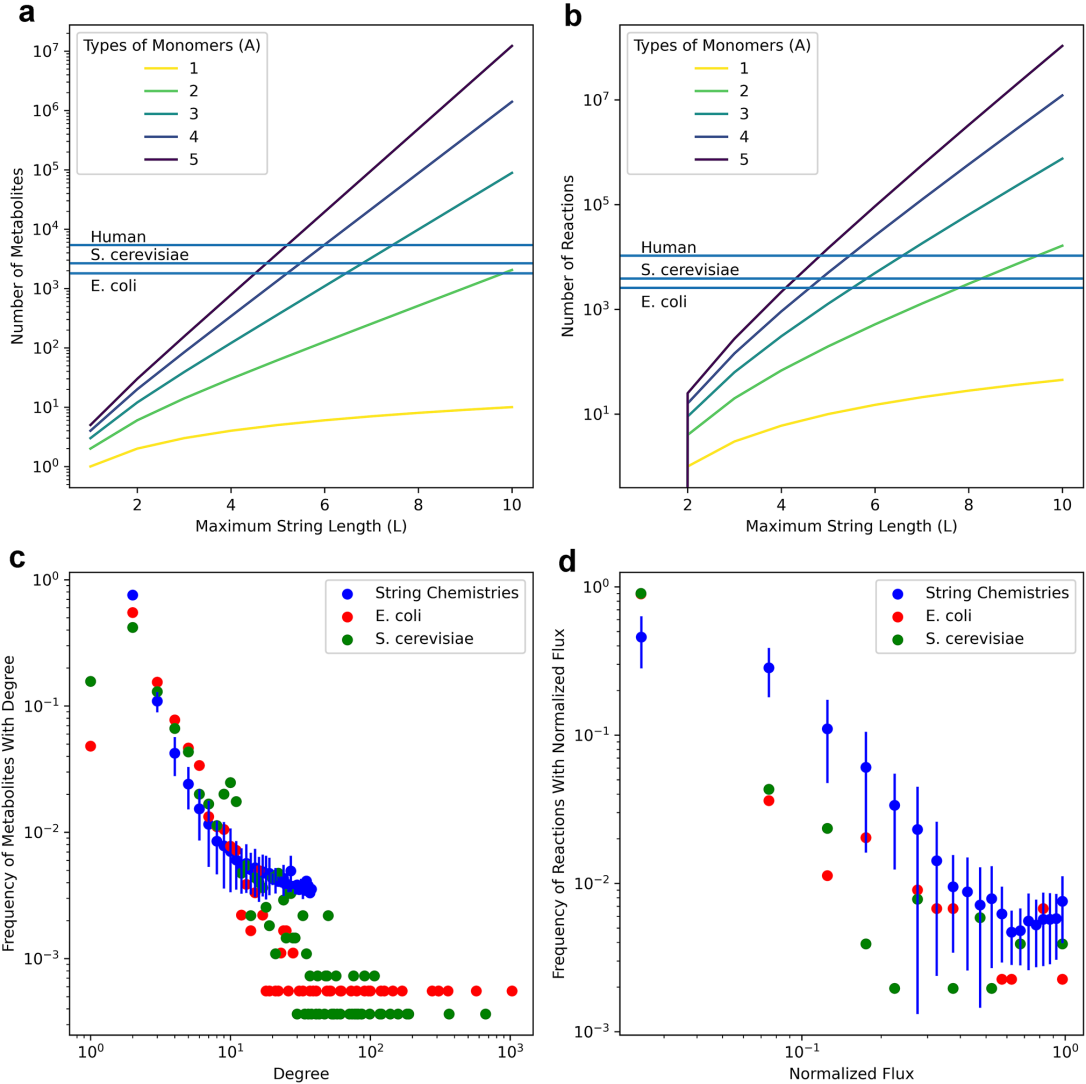
Comparison of size, degree distributions, and flux distributions of string chemistry networks to the same properties of real metabolic networks. **a** Network sizes of universal string chemistry networks (colored lines) and real metabolic networks (blue lines) measured by metabolite counts. **b** Network sizes measured by reaction counts. **c** Degree distributions of 100 string chemistry networks pruned from the universal network with *A* = 3 and *L* = 7 compared to degree distributions of real metabolic networks (see “Methods” section for more details). String chemistry degree distributions are shown as mean and standard deviations of frequencies of each degree across the 100 pruned networks. **d** Flux distributions of 100 string chemistry networks (same as in **c**) compared to flux distributions of real metabolic networks when optimized with the default biomass objective functions (see “Methods” section for more details). Fluxes were normalized to the maximum flux within each network and binned into 10 equally sized bins before plotting. Flux distributions of string chemistry networks are shown as mean and standard deviations of frequencies across the 100 pruned networks

The artificial chemistry model used here is an extension of the one used in Riehl et al. (2010) and is similar to several other previously used artificial chemistries (Banzhaf and Yamamoto 2015; Kauffman 1993; Fontana and Buss 1994a, b): each “chemical” is a string of characters of some arbitrary length, where each character represents an individual atom (or functional group, or monomer). A chemical may condense with one other chemical to produce a longer chemical; the two strings are simply concatenated (e.g., ab + aa → abaa). A chemical may also split into two smaller chemicals at any point along its length (e.g., ababb → ab + abb). Only pairwise condensation/dissociation reactions were considered due to the rarity of termolecular and higher reactions in real chemistry (Chang 2005; Laidler and Glasstone 1948; Compton et al. 2012). For simplicity, all reactions are modeled as being completely reversible, even though in principle further constraints on reversibility could easily be added. The numbers of chemicals and allowed reactions in the model are functions of the number of unique characters (“monomers”) and the maximum chemical length. These functions, plotted in Fig. 2, can be obtained analytically by enumerating the sizes of various string chemistry networks and examining the resulting series:

$$\# \;{\text{metabolites}} = \sum\limits_{{i = 1}}^{L} {A^{i} = \left\{ {\begin{array}{*{20}l} {\frac{{A(A^{L} - 1)}}{{(A - 1)}}} & {A \ne 1} \\ L & {A = 1} \\ \end{array} } \right.} ,$$

$$\# \;{\text{reactions}} = \sum\limits_{{i = 1}}^{L} {(i - 1)A^{i} = \left\{ {\begin{array}{*{20}l} {A\frac{{(L - 1)A^{{L + 1}} - LA^{L} + A}}{{(A - 1)^{2} }}} & {A \ne 1} \\ {\frac{{L(L - 1)}}{2}} & {A = 1} \\ \end{array} } \right.}$$

where *A* is the number of unique characters (monomers) and *L* is the maximum chemical length. We will refer below to a specific complete set of metabolites and reactions generated for a given choice of *A* and *L* as a “chemical universe”. This will allow us to clearly distinguish such complete sets from subsets generated by pruning algorithms (see below).

### Flux Balance Analysis

**Fig. 3:**
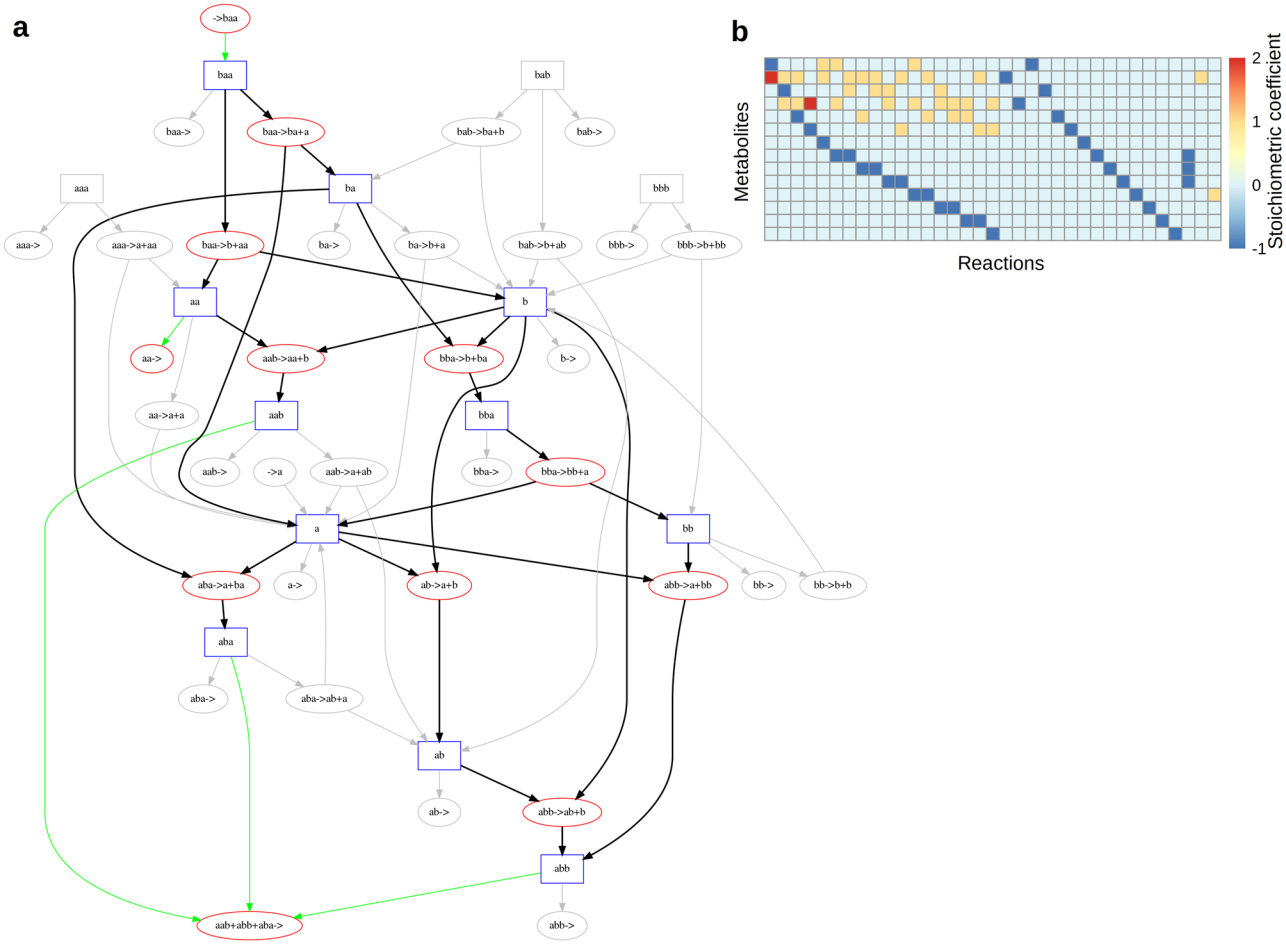
Flux Balance Analysis on string chemistry networks. **a** String chemistry network with *A* = 2 and *L* = 3. Metabolites are represented by blue rectangles and reactions are represented by red ovals. Edge colors represent reaction fluxes after maximizing flux through the biomass reaction: green edges are exchange fluxes (import/export/biomass production), black edges represent nonzero fluxes, and gray edges represent fluxes of zero. The direction of non-gray edges corresponds to the direction of flux; directions on gray edges are arbitrary. **b** Stoichiometric matrix of network in (**a**). Each entry in the matrix represents the stoichiometric coefficient of a particular metabolite (row) in a particular reaction (column), and the coefficient is positive if the metabolite is produced by the reaction or negative if the metabolite is consumed by the reaction

Flux Balance Analysis (FBA) is a mathematical framework for computing steady-state fluxes through chemical reactions in a given network of reactions subject to linear constraints (Orth et al. 2010). The network of reactions is represented as a stoichiometric matrix **S**, where each column represents an individual reaction and each row represents an individual metabolite. Each element in this matrix is the stoichiometric coefficient for the given metabolite in the given reaction: positive if the metabolite is a product of that reaction, negative if it is a substrate, and zero if it does not participate (see Fig. 3 for an example of a string chemistry network and its associated stoichiometric matrix). The reaction fluxes to be computed are represented by a vector **v**. In order for the network to be at steady state, **v** must be in the null space of **S**

$$\user2{Sv} = 0$$

The resulting system of equations is underdetermined for nearly all nontrivial networks. Additional constraints may be specified that limit the values of fluxes through specific reactions, typically reflecting nutrient limitations or known thermodynamic constraints on certain reactions. These constraints generally reduce the space of feasible solutions, but still leave the problem underdetermined. Thus, a linear combination of reactions **Z** (the objective function) through which flux should be maximized (or minimized) is also specified:

$$\user2{Z} = \user2{c}^{{\text{T}}} \user2{v}$$

where **c** is a vector indicating which reactions are to be included in the objective function **Z**. As FBA is usually applied to biochemical reaction networks, the objective function is frequently set to correspond to a single reaction that produces the right proportion of all precursors necessary for the generation of cellular macromolecules and key metabolites, representing growth of cellular biomass. While FBA was originally developed for studying and engineering microbial metabolic networks, its formalism is easily adaptable to any chemistry, provided that its chemical reactions can be represented as columns of a stoichiometric matrix (Fig. 3).

### The ARtificial CHemistry NEtwork Toolbox (ARCHNET) Package

We created the ARCHNET Python package to facilitate the creation and handling of string chemistry networks (as defined above) of arbitrary size, as well as the application of FBA to such networks. All FBA computations were performed using the COBRApy Python package (Ebrahim et al. 2013). The ARCHNET package, along with all scripts used to generate data and create figures, is available in a GitHub repository: https://github.com/segrelab/string-chemistry. The package contains tools for generating and analyzing string chemistry networks of arbitrary size, given the set of characters to use as monomers and the maximum string length. A network can be returned as a stoichiometric matrix and/or a COBRApy model (to facilitate applying FBA or any other stoichiometric modeling technique).

### Network Pruning Algorithm

We implemented an algorithm that takes a complete string chemistry network as an input (e.g., the network of all possible reactions and metabolites when *A* = 2 and *L* = 5) and outputs a subnetwork that has been pruned to satisfy specific criteria. Specifically, the algorithm takes as input (i) a string chemistry network, (ii) a set of available environmental nutrients, and (iii) a biomass composition, i.e., a set of molecules that have to be produced at stoichiometrically fixed proportions; in all examples shown here, we use coefficients of 1 each for molecular components of biomass, except in Fig. S6. In principle, however, biomass coefficients could have any empirically assigned value, as done in the stoichiometric models of real organisms, where these numbers represent the amount in millimoles of that molecule per 1 g of biomass. The algorithm iteratively removes reactions from the network until there is no flux through the output reaction (Fig. S1). In particular, it repeatedly runs FBA to assign fluxes to all reactions and removes reactions with no flux and the reaction with the smallest nonzero flux. Once there is no flux through the output reaction, the last reaction that was removed is added back to the network and the network is “pruned”. The pruning algorithms are part of the Python package described above. Several other assorted scripts provide examples of applications of this pruning algorithm to string chemistry networks.

### Comparing Degree and Flux Distributions of Metabolic Networks

In order to compare the degree distributions of string chemistry networks and real metabolic networks, COBRApy models of string chemistry networks, iJO1366 (*Escherichia coli*, Orth et al. 2010), and Yeast8 (*Saccharomyces cerevisiae* Lu et al. 2019) were used to create graph representations of those metabolic networks using the networkx package (https://github.com/networkx/networkx). The networks were represented as bipartite graphs, where each node represents either a metabolite or a reaction, and two nodes are connected when a metabolite participates in a reaction (either as a product or reactant; edges are undirected). When computing degree distributions, only the degrees of metabolite nodes were considered.

Flux distributions were generated for all string chemistry networks and real metabolic networks by performing FBA with the default biomass objective functions. In order to facilitate comparison of fluxes between different networks, all fluxes within each network were normalized to the largest flux in the network (after taking the absolute value of all fluxes), and fluxes were binned.

## Results

### A Python Package for Creating and Analyzing Arbitrary String Chemistries

We have created the ARtificial CHemistry NEtwork Toolbox (ARCHNET), a Python package capable of generating string chemistry networks of arbitrary sizes given the number of unique characters (*A*) and the maximum length of a string (*L*) (Fig. 1). For simplicity, the only types of reactions allowed in these networks are pairwise string concatenation and splitting (see “Methods” section for more details). Even with this restriction on reaction complexity, the networks increase in size very rapidly as *A* and/or *L* increase (Fig. 2a, b and “Methods” section). For example, a basic chemistry with *A* = 3 and *L* = 2 would have 12 metabolites and 9 reactions. If we increase *A* by 1, the network would involve 20 metabolites and 16 reactions. If we instead increased *L* by one, there would be 39 metabolites and 63 reactions. The network sizes therefore depend very differently on these two parameters (see “Methods” section). One of the important features of the package is that it can output networks both as a simple text file containing the stoichiometric matrix, and as a COBRApy model (Ebrahim et al. 2013), which can be exported as an SMBL file (Hucka et al. 2003) used in most tools developed to study real metabolic networks, including standard FBA calculations (Fig. 3).

While the principles constraining the structure of real metabolic networks are much more complicated than those giving rise to our string chemistry networks, string chemistry networks (of equal or lower complexity) can still reproduce some network-level properties of real chemistry networks (Riehl et al. 2010). As shown in Fig. 2a, b, specific sets of parameters in artificial chemistries can lead to networks that contain numbers of reactions and metabolites that are close to those of real metabolic networks. These numbers can be determined either numerically or analytically (see “Methods” section). Interestingly, even string chemistry networks with few unique characters and short maximum lengths (e.g., *A* = 4, *L* = 5; *A* = 2, *L* = 10) reach sizes comparable to those of the human, yeast and *E. coli* metabolic networks (Fig. 2a, b; Orth et al. 2010; Lu et al. 2019). However, as seen in Fig. S2, these artificial networks have a much higher connectivity (ratio of reactions to metabolites) than the real organisms’ metabolic networks. Conversely, a simple network with *A* = 1 and *L* = 3 would have a connectivity comparable to that of real metabolic networks, but would be much smaller. One could then ask whether it is possible to create string chemistry networks that are both of similar sizes and connectivities to those of real metabolic networks. Indeed, one should view the complete string chemistries depicted here as analogous to “complete chemical universes”, out of which a single organism’s metabolic network would constitute a small subset. As shown below, this concept can be explored in artificial chemistries by devising algorithms that can “prune” complete chemical networks to obtain subnetworks that resemble individual organisms’ metabolic networks.

### Pruned Networks as Proxies for Evolved Organisms

Having established a method for quantitatively comparing properties of string chemistry networks to real metabolic networks, we explored the properties of string chemistry subnetworks that more closely resemble those of individual organisms. We thus modeled organism-scale metabolic networks as “minimal networks,” which use the fewest reactions required to produce a desired set of metabolites (i.e., biomass precursors, in analogy with the building blocks of microbial biomass used to represent self-reproduction in the growth flux associated with genome-scale stoichiometric models (Orth et al. 2010; Lachance et al. 2019)). This minimal network structure is consistent with simple parsimonious evolutionary assumptions used in previous studies (Noor et al. 2010; Riehl et al. 2010; Pál et al. 2006). To identify these minimal networks from our string chemical universes generated using ARCHNET, we implemented a “pruning” algorithm that iteratively applies FBA to string chemistry networks. Briefly, the algorithm works by (1) applying FBA to a string chemistry network (initially set to the whole chemical universe given particular values of *A* and *L*) with some specified nutrient uptake reactions and a “biomass” reaction (representing the metabolic objective of the network, e.g., biomass production for many real metabolic networks), (2) removing all reactions that have no flux, (3) testing whether or not the reaction with the smallest nonzero flux can be removed without eliminating flux through the biomass reaction, and (4) repeating until no reactions can be removed (see “Methods” section and Fig. S1).

One may wonder whether the structures of these pruned networks resembles those of real organisms’ metabolic networks. We addressed this question by computing degree and flux distributions for 100 pruned networks (*A* = 3, *L* = 7) and comparing them to the degree and flux distributions of the *E. coli* and *S. cerevisiae* metabolic networks (see “Methods” section). As shown in Fig. 2c, the pruned string chemistry networks tend to have scale-free degree distributions, just as the real metabolic networks do (Almaas et al. 2004). As shown in Cohen et al. (2004), scale-free networks are also necessarily small-world networks, and real metabolic networks are well known to be small-world networks (Wagner and Fell 2001). The distributions of fluxes through the pruned string chemistry networks strongly resemble the distributions of fluxes through the *E. coli* and *S. cerevisiae* networks when optimized for biomass production (Fig. 2d).

Given these structural similarities, we asked whether pruned networks could also reflect some of the functional properties of real networks. We looked specifically at metabolic secretions, as the ability to excrete waste products is a crucial component of cellular metabolism (Ferguson et al. 1998; Richards et al. 2013; Hart et al. 2019). Since the degree to which individual bacteria secrete metabolic intermediates and/or waste products varies dramatically from organism to organism, we examined two extreme cases with our pruning algorithm: one in which all metabolites in the network are allowed to be secreted, and one in which no metabolites are allowed to be secreted (i.e., the only sink in the network is the biomass reaction). We found that pruning while allowing secretion of waste products generally resulted in slightly smaller networks than pruning without allowing secretion (Fig. S2). This is likely because ensuring that all metabolic byproducts are internally recycled into biomass components requires more reactions than simply secreting them as waste products. We also note that the notion that secreting waste products leads to simpler metabolic networks may well be relevant in real microbial communities. For example, many microbial communities are sustained by costless secretions (Pacheco et al. 2019), which could lead to a reduction of metabolic capabilities in specific taxa (Morris et al. 2012).

### Biomass Precursors Shape Network Composition More than Environmental Nutrient Composition

**Fig. 4:**
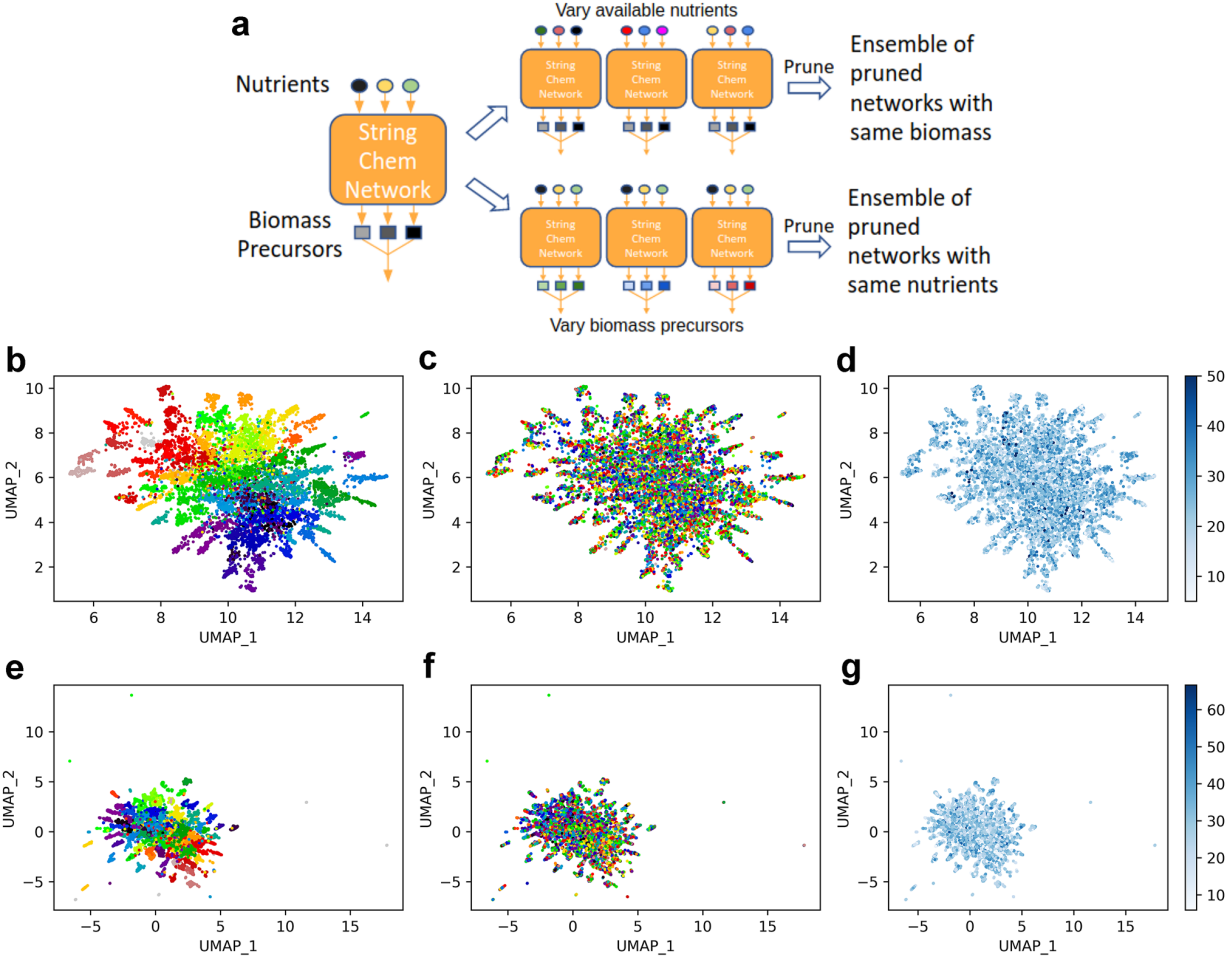
Choice of biomass precursors impacts structure of pruned networks more than choice of available nutrients. **a** Cartoon representation of how data shown in panels (**b**–**g**) was generated. **b** UMAP scatterplot of pruned networks with export reactions (see main text) generated as described in (**a**). Each point represents a different pruned network and the color of each point indicates the biomass reaction of that network. **c** Same as (**b**) but colors indicate which set of nutrients the network was pruned with. **d** Same as (**b**) but colors indicate optimal biomass flux. **e**–**g** Same as (**b**–**d**) but networks were pruned without export reactions (see main text). All pruned networks were derived from the universal string chemistry network with *A* = 2 and *L* = 5

Using our pruning algorithm, we investigated the relative importance of the choice of nutrients and the choice of biomass precursors on the composition (i.e., identities of remaining reactions) of pruned networks. We generated a string chemistry universe with *A* = 2 and *L* = 5, then created different biomass compositions (100 different sets of 5 randomly chosen biomass precursors) and different sets of nutrients (100 random pairs of nutrients) using the metabolites contained within this chemical universe. We note that upon choosing the biomass composition, a biomass reaction was added to produce all chosen biomass precursors in equal proportions (see “Methods” section). We then ran the pruning algorithm on all possible combinations of these nutrients and biomass precursors (Fig. 4a). In order to compare the compositions of the pruned networks, each network was represented as a binary vector with as many elements as there were reactions in the chemical universe. In this binary vector, a 1 represents a reaction that was kept in the pruned network and a 0 represents a reaction that was removed during pruning. These binary vectors were visualized on UMAP plots (McInnes et al. 2018) (Fig. 4b–g). The main outcome of this analysis is that, regardless of whether or not export reactions are allowed, networks with the same biomass typically cluster together (Fig. 4b), while networks with the same nutrients frequently have very different compositions (Fig. 4c). The clustering is generally weaker in the networks pruned without export reactions—there are more isolated networks and distinct small clusters—but the pruned networks still noticeably cluster by biomass reaction (Fig. 4e, f). We also note that the clustering of networks does not seem to display any clear pattern in terms of achievable growth rates (Fig. 4d, g), which are highly variable and roughly distributed around an intermediate value. In other words, networks with similar composition, as dictated by the biomass composition, may achieve substantially different growth rates, suggesting that while biomass composition dictates network composition, growth rates are not as straightforwardly determined by either biomass composition or environmental composition.

To assess the possibility that these results were an artifact of the arbitrarily chosen number of nutrients and biomass precursors, we investigated how the proportion of pruned reactions and connectivity of pruned networks change as the numbers of nutrients and biomass precursors vary (Figs. S4 and S5, respectively). While the proportion of pruned reactions clearly decreases as the number of biomass precursors increases, as one might expect, it does not appear to be affected by the number of available nutrients. Figure S5 indicates that the metabolite-to-reaction ratio is always around 1 in pruned networks. These values are all slightly lower than those observed in real metabolic networks (Fig. S2), which likely reflects the fact that real metabolic networks must be capable of sustaining growth in multiple different environments, while the pruned string chemistry networks are only required to sustain growth in one particular environment. While we expect that the number of biomass precursors and nutrients may affect the composition of pruned networks in other more subtle ways, these findings support the idea that the results shown in Fig. 4 do not depend on the number of biomass precursors or nutrients used during the pruning process.

## Discussion

We have created ARCHNET, a Python package capable of performing stoichiometric modeling on string chemistry networks of arbitrary size and monomer diversity. We have also devised a pruning algorithm for these networks, which identifies minimal metabolic networks necessary for converting a given set of environmental nutrients into a specific combination of biomass precursors. By applying this pruning algorithm to many thousands of string chemistry networks, we found that the choice of the biomass metabolites wields much more influence over the composition of the minimal network than the choice of environmental nutrients. Beyond this finding, our package could be used to further quantitatively explore any aspect of the complex relationship between metabolic network structure, environmental diversity, and biomass composition.

It is important to keep in mind that while in the current work we verified the robustness of some of our results relative to a number of free parameters, there are additional choices of the underlying string chemistry and pruning algorithm that could influence the results. In particular, as described above, and shown in Figs. S4 and S5, we verified that pruned network properties depend only mildly on the choice of the number of biomass components and nutrients, suggesting that downstream analyses would similarly be robust relative to these two parameters. When generating the pruned networks shown in Fig. 4, all stoichiometric coefficients used in all involved biomass reactions were 1, so in Fig. S6 we verified that varying the values of these stoichiometric coefficients does not significantly alter the main conclusions drawn from Fig. 4. To generate the pruned networks in Fig. S6, the ensembles of networks with identical biomass reactions and varied nutrients (see Fig. 4a for a schematic) underwent one additional modification before pruning: each stoichiometric coefficient in each network’s biomass reaction was changed from 1 to a random integer between 1 and 10 (inclusive). As shown in Figure S6a, pruned networks with the same biomass precursors (but not necessarily the same stoichiometric coefficients) cluster together to a comparable extent as the pruned networks in Fig. 4b. The pruned networks do not seem to cluster by nutrient sources in either Figs 4c, f or S6b. No particular sets of biomass precursors seem to result in consistently higher or lower maximum growth fluxes than any other sets (Figs. 4d, g and S6c). Further analyses may uncover more subtle ways in which the particular values used in the stoichiometric coefficients of biomass reactions relate to the structures of pruned networks.

There are also a number of ways in which the string chemistry model presented here could be made more complex and/or realistic. One could try to explicitly model more features of real chemical reactions (e.g., thermodynamics, kinetics), along the lines of the methods mentioned in the introduction. The role of reaction irreversibility represents a key area of future exploration: while all internal reactions are currently assumed to be perfectly reversible, variable degrees of reaction reversibility (e.g., induced by free energy assignments) could be added to string chemistries to recapitulate features observed in real metabolism. One could also introduce regulatory interactions between metabolites and reactions by connecting the fluxes through particular metabolites to the constraints on fluxes through other reactions. Notably, while each of these possible modifications would not necessarily dramatically change the string chemistry framework, the combinatorial interactions of all the various new parameters would dramatically increase the space of possible networks to explore and characterize, providing ample opportunities for future work with artificial chemistries.

We believe that our finding about the relative importance of biomass composition and environmental metabolites has important consequences for the process of reconstructing real metabolic networks. A key step in the process of creating Genome-Scale Metabolic Models (GEMs) is the process of gap filling, where missing reactions (“gaps”, usually due to incomplete experimental data) in draft GEMs are imputed using a variety of methods (Thiele et al. 2014; Satish Kumar et al. 2007; Prigent et al. 2017; Christian et al. 2009; Vitkin and Shlomi 2012). Gap-filling algorithms generally function by identifying a minimal set of reactions to add to the draft GEM in order for it to be capable of producing biomass, so they require users to specify a particular biomass reaction. Frequently, the so-called “template” biomass reactions are used for all bacteria in a particular taxa (e.g., many GEMs of Gram-negative bacteria are created using the *E. coli* biomass reaction) due to the significant difficulty of obtaining the extensive experimental data required to determine a particular organism’s biomass composition (Henry et al. 2010; Xavier et al. 2017). Our finding about the role biomass composition plays in determining the composition of pruned networks, along with a previous study that found that bacterial GEMs clustered more strongly by which biomass reaction was used as a template than by taxonomy (Xavier et al. 2017) both suggest that careful consideration should be put into the choice of a biomass reaction when using gap-filling algorithms.

The pruning algorithms presented in this work bear some resemblance to certain gap-filling algorithms, algorithms for identifying Elementary Flux Modes (EFMs) (Schuster et al. 2000), and algorithms for identifying Minimal Balance Pathways (MBPs) (Riehl et al. 2010). Some gap-filling algorithms iteratively add and remove reactions from a large pool of possible reactions, eventually converging to an optimally gap-filled network (Vitkin and Shlomi 2012; Reed et al. 2006; Pharkya et al. 2004). Some algorithms for deriving tissue-specific metabolic networks from generic human metabolic networks also function similarly (Machado et al. 2018; Jerby et al. 2010). EFMs represent every independent route from a source to a sink through a metabolic network and are often considered a basis set for the space of possible fluxes through a network; the pruned networks we present here may converge to EFMs of string chemistry networks for certain input/output metabolite cases, but further research may identify additional connections between the two concepts. Since MBPs represent the optimal set of reactions for converting a single input metabolite into a single output metabolite, our pruned networks could be viewed as an extension of MBPs with multiple inputs and multiple outputs. All of these algorithms, including our pruning algorithms, aim to identify “optimal” networks under certain criteria of optimality; further exploration of the similarities and differences between these approaches may lead to a better understanding of what one should consider an optimal metabolic network to be like.

The pruning algorithm presented in this paper is far from the only algorithm that accomplishes the goal of narrowing down a metabolic network to its essential components, given an environment and a biomass composition. In an attempt to verify that the precise details of how our pruning algorithm was formulated do not substantially impact the main results of this work, we created an alternate pruning algorithm: instead of removing the reaction with the smallest flux, the new pruning algorithm computes the change in biomass flux that would result from every possible single reaction deletion and removes the reaction with the smallest impact on biomass flux at each pruning step. The two pruning algorithms generally remove the same reactions at each step of the algorithm (Fig. S7) and usually wind up producing very similar output networks when given the same input (Fig. S8). Furthermore, when reproducing the analysis shown in Fig. 4a with the biomass-focused pruning algorithm, the results are entirely comparable to those obtained using the original pruning algorithm (Fig. S9). Based on these analyses, we believe that the specific criterion used to decide which reactions should be pruned do not meaningfully change the main conclusions of this paper.

One can view both of these pruning algorithms as analogs of reductive evolutionary processes: the flux-based pruning algorithm selects against reactions that carry little flux, while the biomass-based pruning algorithm selects against reactions that contribute little to biomass production. One could imagine that a microbe growing in a nutrient-poor environment might stop devoting resources to expressing metabolic enzymes that catalyze reactions that carry little flux and are not essential for biomass production. Similarly, one could imagine a competitive environment in which an organism capable of achieving similar growth rates to its neighbors while devoting fewer resources to producing metabolic enzymes that do not substantially contribute to biomass production would outcompete its neighbors. The pruned networks are also reminiscent of the metabolic networks of certain marine plankton species that only express half of the enzymes in the citric acid cycle, since other microbes in their environment secrete the appropriate intermediates and there is strong selective pressure to reduce genome size due to low availability of nitrogen (Braakman et al. 2017). While the outputs of both pruning algorithms are generally similar, there is a small subset of initial networks that are pruned rather differently by the two algorithms (Fig. S8); studying these cases in more detail may yield new insights into general features of these different types of evolutionary processes.

Several previous studies used artificial chemistry as an avenue for addressing questions related to the origin of life or to general mathematical properties of biochemical networks (Banzhaf and Yamamoto 2015; Kauffman 1993; Benkö et al. 2003; Walter Fontana and Buss 1994a, b; Peng et al. 2020). Conversely, FBA has been applied mostly to the study of metabolic networks of real organisms (Gu et al. 2019; Kauffman et al. 2003; Orth et al. 2010). There is likely great untapped potential available from combining the two approaches. In particular, the recent application of stoichiometric approaches to the study of early metabolism (Goldford and Segrè 2018) and of ecosystem-level biochemical networks (Carlson et al. 2018; Klitgord and Segrè 2010; Harcombe et al. 2014) could greatly benefit from additional creative usage of artificial chemistries. For example, the capacity to handle artificial string chemistries of arbitrary complexity using these same stoichiometric tools makes it possible to explore evolutionary processes and ecosystem-level metabolism under simulated scenarios in which the whole chemical universe is fully known. One could create an assortment of string chemistry networks using ARCHNET and model their interactions using tools such as COMETS (Dukovski et al. 2020). This will make it possible to shed light on the role of historical contingency and optimality principles in shaping the structure of metabolic networks.

## Supplementary Information

Below is the link to the electronic supplementary material.

Fig. S1 Description of the pruning algorithm. Supplementary file1 (PNG 29 kb)

Fig. S2 Comparison of string chemistry networks (colored lines) to real metabolic networks (black lines) using ratio of reactions to metabolites. Ratios for networks pruned from the chemical universe with A = 2 and L = 5 are shown as a boxplot. Supplementary file2 (PNG 539 kb)

Fig. S3 Pruning while allowing export reactions results in slightly smaller networks. The chemical universe where A = 2 and L = 5 was pruned on 100 random combinations of 2 nutrients and 5 biomass precursors and the sizes of the pruned networks were recorded as a number of metabolites in the network and b number of reactions in the network. Supplementary file3 (PNG 388 kb)

Fig. S4 Number of biomass precursors affects the percentage of pruned reactions more than the number of available nutrients. The chemical universe where A = 2 and L = 5 was pruned with 100 different combinations of each number of food sources and biomass precursors shown on the graph. Each point is the mean pruned percentage with error bars indicating the standard deviation. Supplementary file4 (PNG 311 kb)

Fig. S5 Ratio of reactions to metabolites in pruned networks remains generally constant as numbers of available nutrients and biomass precursors are varied. All networks were pruned from the chemical universe where A = 2 and L = 5 with export reactions allowed. Each point is the mean reaction-to-metabolite ratio with error bars indicating the standard deviation. Supplementary file5 (PNG 327 kb)

Fig. S6 Altering stoichiometric coefficients in biomass reactions reproduces phenomenon where choice of biomass precursors impacts structure of pruned networks more than choice of available nutrients. a UMAP scatterplot of pruned network generated as described in Figure 4a with one extra step: after generating ensembles of networks with identical biomass precursors and different nutrients but before pruning, change all stoichiometric coefficients in the biomass reactions to random integers between 1 and 10 (inclusive). Each point represents a different pruned network and the color indicates which set of biomass precursors were used in that network’s biomass reaction. b Same as a but colors indicate which set of nutrients the network was pruned with. c Same as a but colors indicate optimal biomass flux. Supplementary file6 (PNG 623 kb)

Fig. S7 The two pruning algorithms generally follow similar trajectories as they remove reactions from identical starting networks. 100 different combinations of 2 nutrients and 5 biomass precursors were randomly selected from the chemical universe where A = 2 and L = 5. Each combination of nutrients and biomass precursors was used as input to both the minimum-flux pruning algorithm and the biomass-impact pruning algorithm (see main text) and the number of reactions in the pruned network at each pruning step was recorded. Supplementary file7 (PNG 1380 kb)

Fig. S8 The two pruning algorithms generally produce similar output networks when provided with identical input networks. 100 different combinations of 2 nutrients and 5 biomass precursors were randomly selected from the chemical universe where A = 2 and L = 5. Each combination of nutrients and biomass precursors was used as input to both the minimum-flux pruning algorithm and the biomass-impact pruning algorithm, and the lists of reactions in each pruned network were recorded. Jaccard similarities were computed between the lists of reactions in each pair of networks pruned from the same initial network. Supplementary file8 (PNG 150 kb)

Fig. S9 Alternative pruning algorithm reproduces phenomenon where choice of biomass precursors impacts structure of pruned networks more than choice of available nutrients. a UMAP scatterplot of pruned networks with export reactions (see main text) generated as described in Figure 4a. Each point represents a different pruned network and the color of each points indicates the biomass reaction of that network. b Same as a but colors indicate which set of nutrients the network was pruned with. c Same as a but colors indicate optimal biomass flux. Supplementary file9 (PNG 2352 kb)

## Data Availability

All codes used to generate data and figures in this work are available at the following GitHub repository: https://github.com/segrelab/string-chemistry
