# Supplementary figures and images for: Stoichiometric Modeling of Artificial String Chemistries Reveals Constraints on Metabolic Network Structure
Source: J Mol Evol. 2021 Jul 6;89(7):472–83. doi: 10.1007/s00239-021-10018-0 (PMC8318951; doi:10.1007/s00239-021-10018-0)

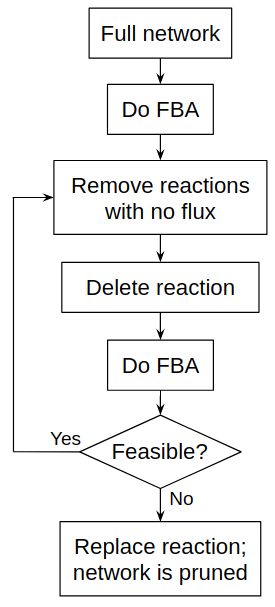

Supplement: Supplementary file 1 — Description of the pruning algorithm. Supplementary file1 (PNG 29 kb) [file 239_2021_10018_MOESM1_ESM.png]

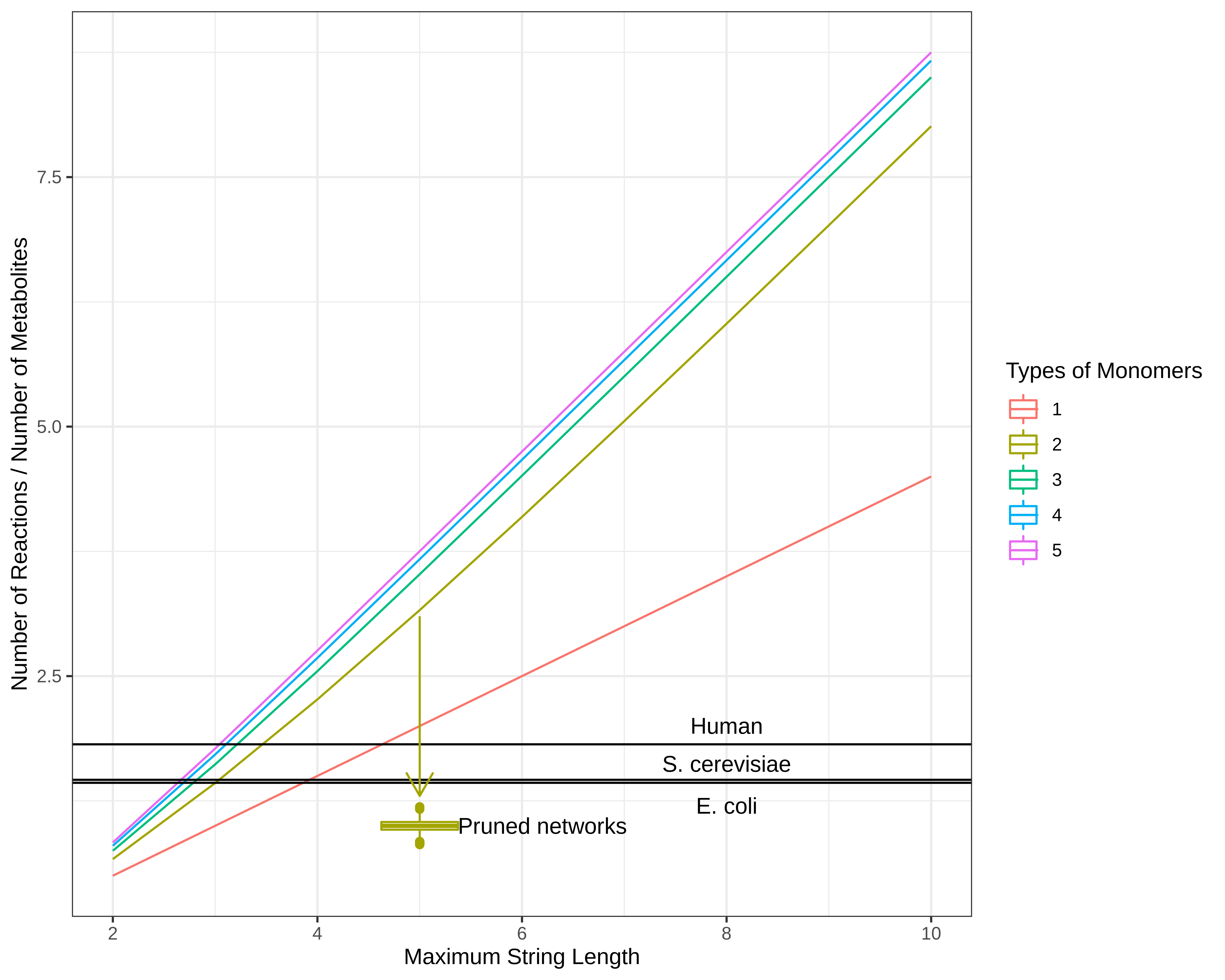

Supplement: Supplementary file 2 — Comparison of string chemistry networks (colored lines) to real metabolic networks (black lines) using ratio of reactions to metabolites. Ratios for networks pruned from the chemical universe with A = 2 and L = 5 are shown as a boxplot. Supplementary file2 (PNG 539 kb) [file 239_2021_10018_MOESM2_ESM.png]

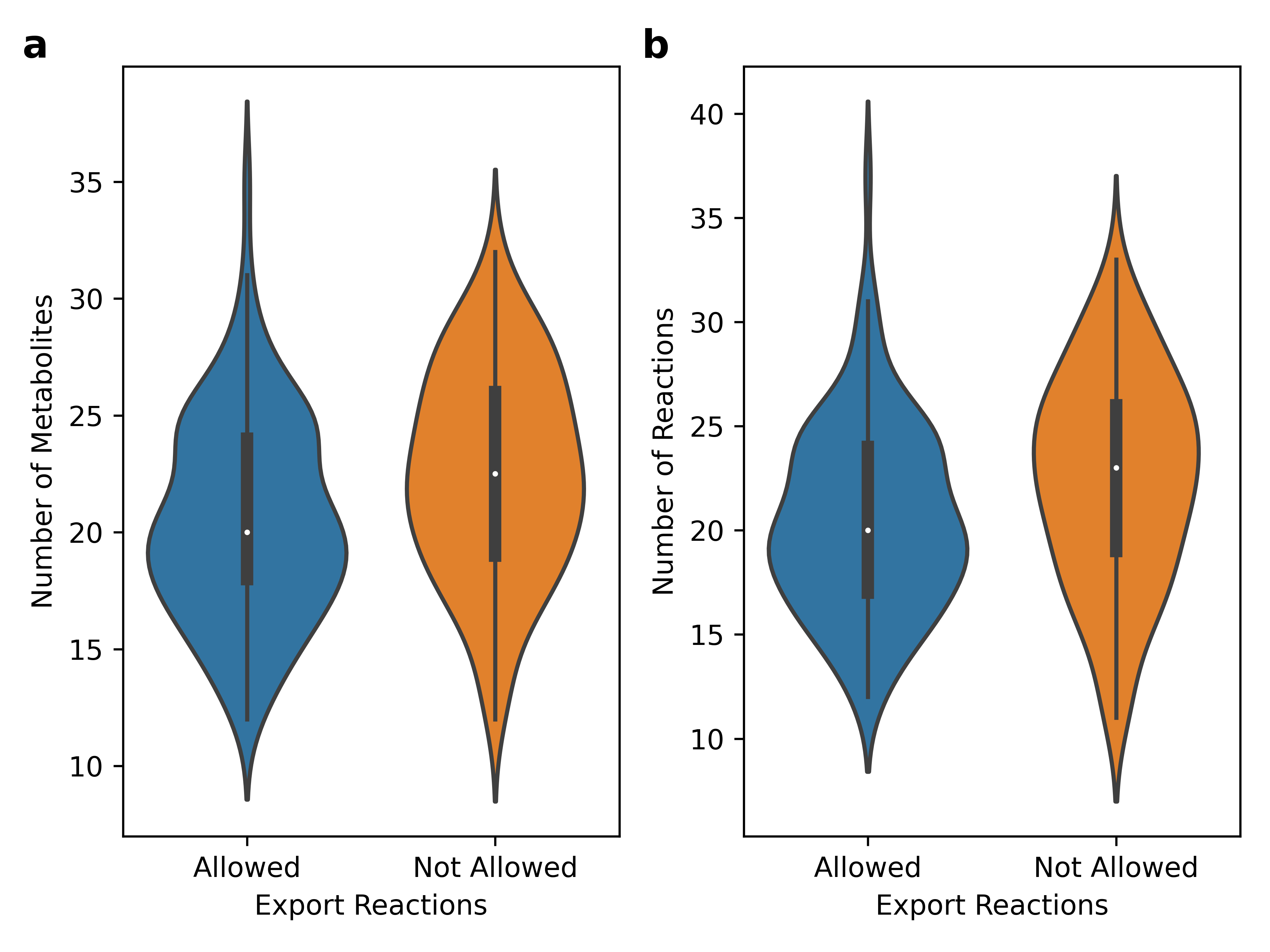

Supplement: Supplementary file 3 — Pruning while allowing export reactions results in slightly smaller networks. The chemical universe where A = 2 and L = 5 was pruned on 100 random combinations of 2 nutrients and 5 biomass precursors and the sizes of the pruned networks were recorded as a number of metabolites in the network and b number of reactions in the network. Supplementary file3 (PNG 388 kb) [file 239_2021_10018_MOESM3_ESM.png]

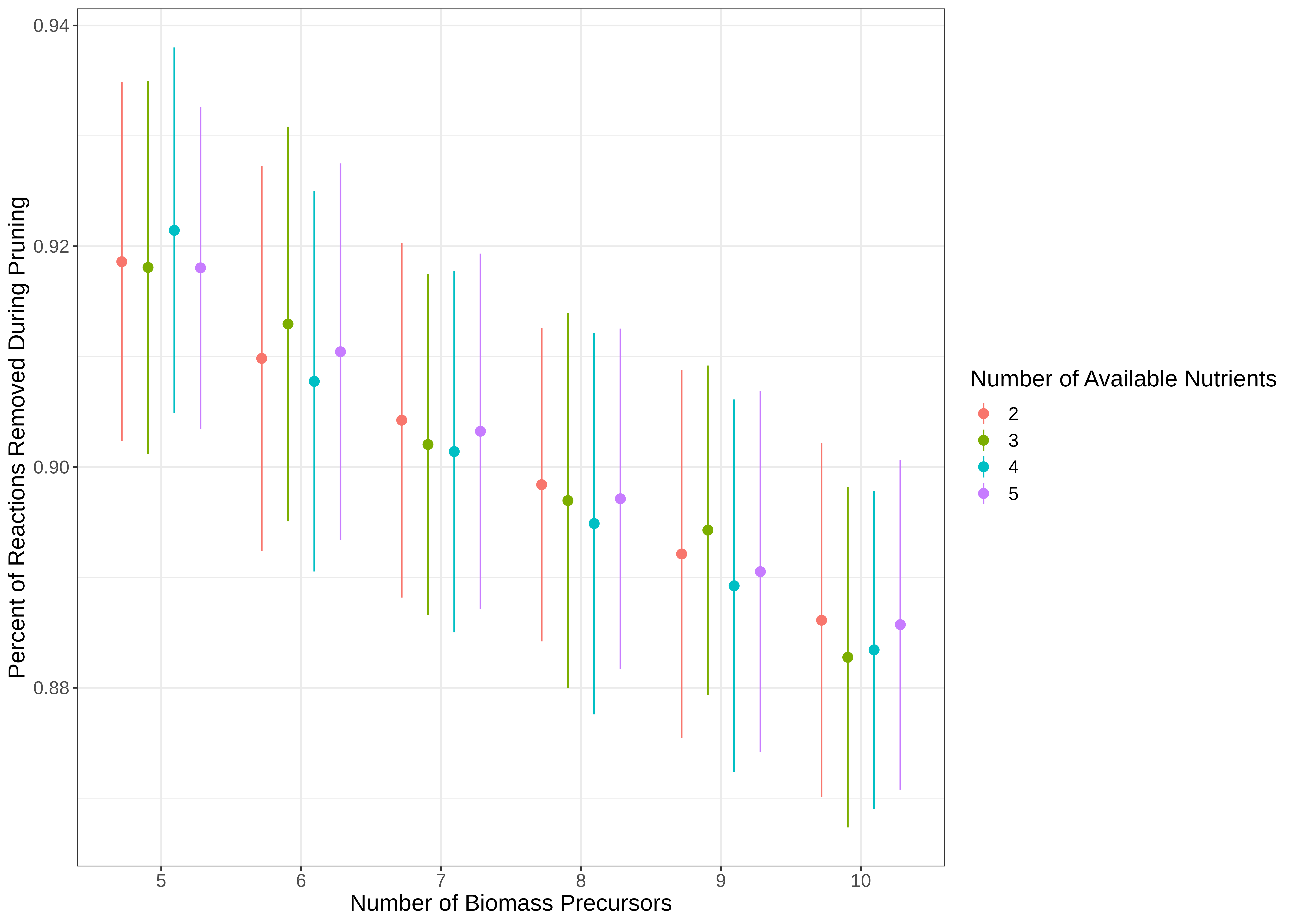

Supplement: Supplementary file 4 — Number of biomass precursors affects the percentage of pruned reactions more than the number of available nutrients. The chemical universe where A = 2 and L = 5 was pruned with 100 different combinations of each number of food sources and biomass precursors shown on the graph. Each point is the mean pruned percentage with error bars indicating the standard deviation. Supplementary file4 (PNG 311 kb) [file 239_2021_10018_MOESM4_ESM.png]

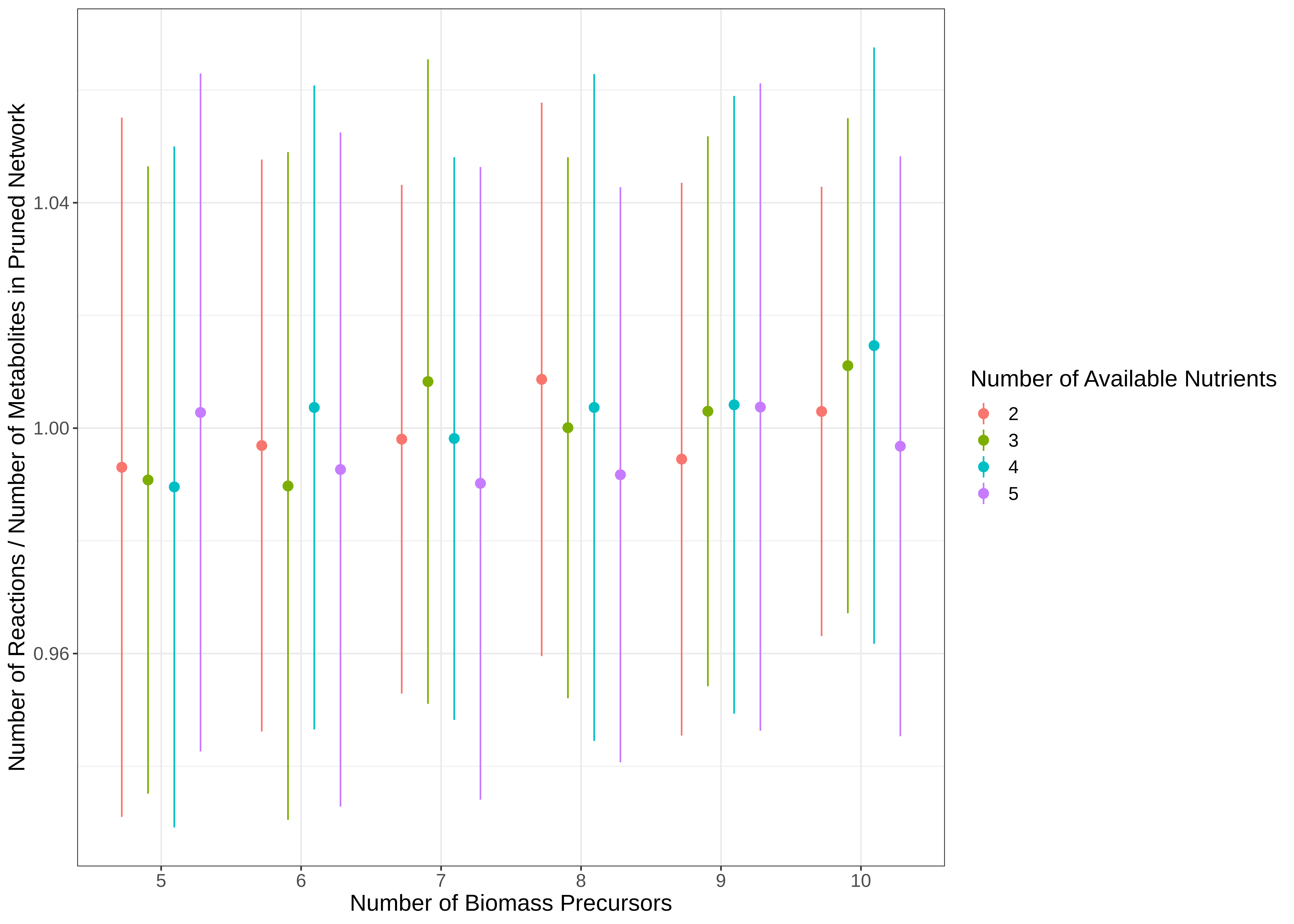

Supplement: Supplementary file 5 — Ratio of reactions to metabolites in pruned networks remains generally constant as numbers of available nutrients and biomass precursors are varied. All networks were pruned from the chemical universe where A = 2 and L = 5 with export reactions allowed. Each point is the mean reaction-to-metabolite ratio with error bars indicating the standard deviation. Supplementary file5 (PNG 327 kb) [file 239_2021_10018_MOESM5_ESM.png]

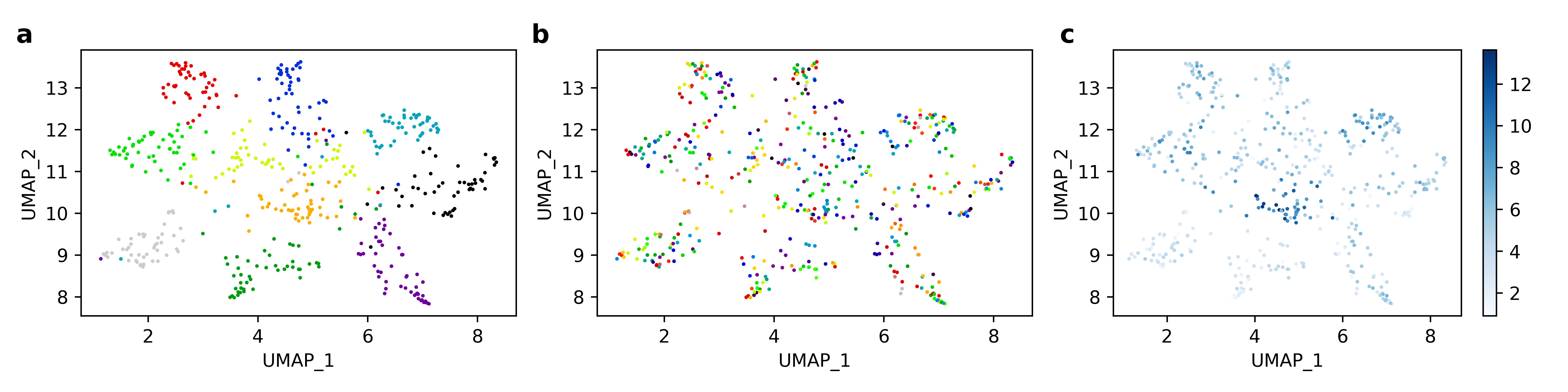

Supplement: Supplementary file 6 — Altering stoichiometric coefficients in biomass reactions reproduces phenomenon where choice of biomass precursors impacts structure of pruned networks more than choice of available nutrients. a UMAP scatterplot of pruned network generated as described in Figure 4a with one extra step: after generating ensembles of networks with identical biomass precursors and different nutrients but before pruning, change all stoichiometric coefficients in the biomass reactions to random integers between 1 and 10 (inclusive). Each point represents a different pruned network and the color indicates which set of biomass precursors were used in that network’s biomass reaction. b Same as a but colors indicate which set of nutrients the network was pruned with. c Same as a but colors indicate optimal biomass flux. Supplementary file6 (PNG 623 kb) [file 239_2021_10018_MOESM6_ESM.png]

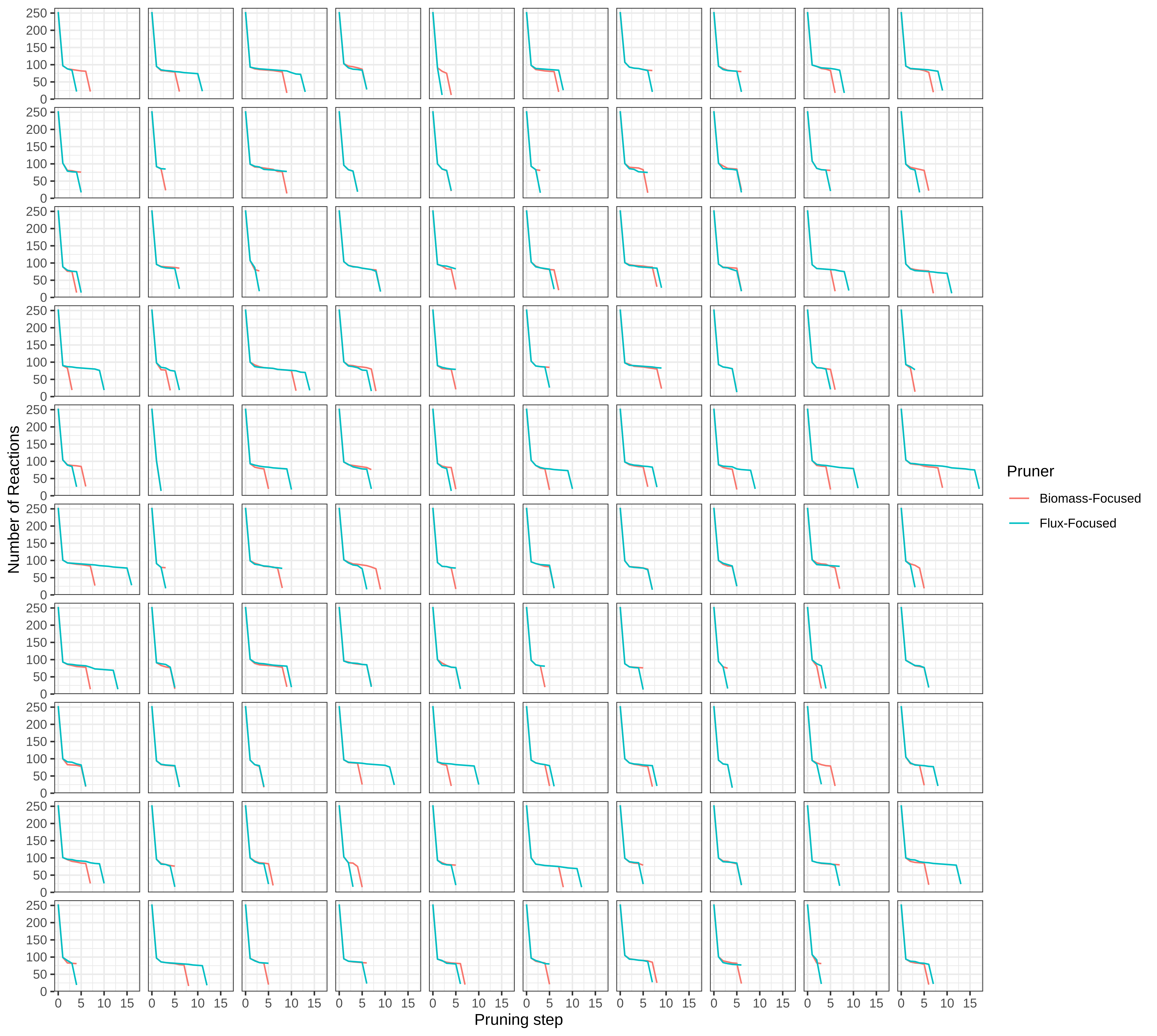

Supplement: Supplementary file 7 — The two pruning algorithms generally follow similar trajectories as they remove reactions from identical starting networks. 100 different combinations of 2 nutrients and 5 biomass precursors were randomly selected from the chemical universe where A = 2 and L = 5. Each combination of nutrients and biomass precursors was used as input to both the minimum-flux pruning algorithm and the biomass-impact pruning algorithm (see main text) and the number of reactions in the pruned network at each pruning step was recorded. Supplementary file7 (PNG 1380 kb) [file 239_2021_10018_MOESM7_ESM.png]

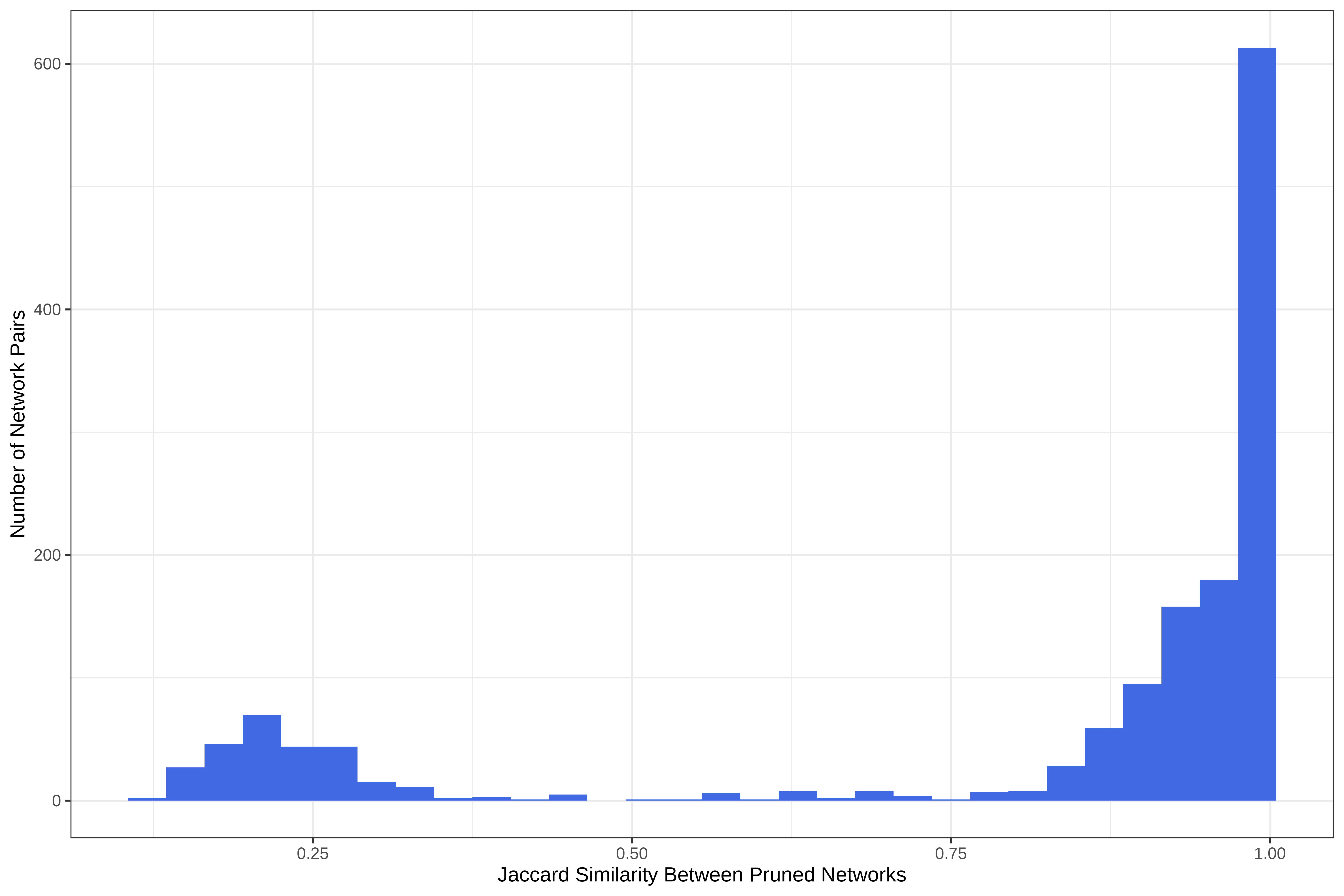

Supplement: Supplementary file 8 — The two pruning algorithms generally produce similar output networks when provided with identical input networks. 100 different combinations of 2 nutrients and 5 biomass precursors were randomly selected from the chemical universe where A = 2 and L = 5. Each combination of nutrients and biomass precursors was used as input to both the minimum-flux pruning algorithm and the biomass-impact pruning algorithm, and the lists of reactions in each pruned network were recorded. Jaccard similarities were computed between the lists of reactions in each pair of networks pruned from the same initial network. Supplementary file8 (PNG 150 kb) [file 239_2021_10018_MOESM8_ESM.png]

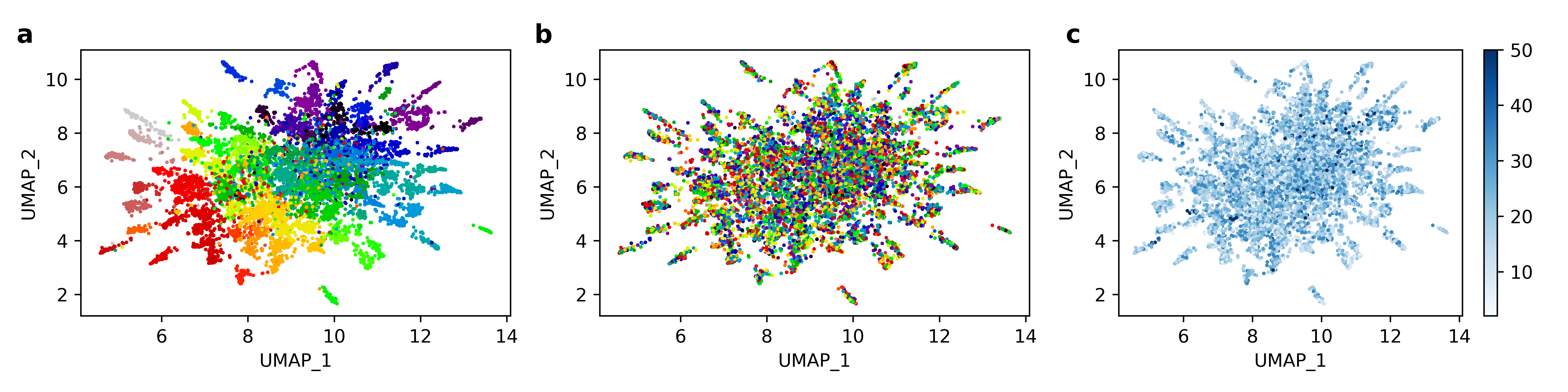

Supplement: Supplementary file 9 — Alternative pruning algorithm reproduces phenomenon where choice of biomass precursors impacts structure of pruned networks more than choice of available nutrients. a UMAP scatterplot of pruned networks with export reactions (see main text) generated as described in Figure 4a. Each point represents a different pruned network and the color of each points indicates the biomass reaction of that network. b Same as a but colors indicate which set of nutrients the network was pruned with. c Same as a but colors indicate optimal biomass flux. Supplementary file9 (PNG 2352 kb) [file 239_2021_10018_MOESM9_ESM.png]
